# Evaluation of the Housing First program in patients with severe mental disorders in France: study protocol for a randomized controlled trial

**DOI:** 10.1186/1745-6215-14-309

**Published:** 2013-09-24

**Authors:** Aurelie Tinland, Cecile Fortanier, Vincent Girard, Christian Laval, Benjamin Videau, Pauline Rhenter, Tim Greacen, Bruno Falissard, Themis Apostolidis, Christophe Lançon, Laurent Boyer, Pascal Auquier

**Affiliations:** 1 Aix-Marseille University, EA 3279 Research Unit, Marseille 13385, France; 2 Department of Psychiatry, Sainte-Marguerite University Hospital, Marseille 13009, France; 3 Laboratoire de Recherche, Etablissement Public de Santé Maison Blanche, Paris, France; 4 INSERM U669, Maison de Solenn, University Paris-Sud and University Paris-Descartes, Paris 75679, France; 5 Aix-Marseille Université, LPS EA849, Aix-en-Provence 13621, France; 6 Department of Research and Innovation, Support Unit for clinical research and economic evaluation, Assistance Publique – Hôpitaux de Marseille, Marseille 13385, France

**Keywords:** Housing first program, Homelessness, Schizophrenia, Bipolar disorder, Mental illness, Evaluation, Randomized controlled trial

## Abstract

**Background:**

Recent studies in North American contexts have suggested that the Housing First model is a promising strategy for providing effective services to homeless people with mental illness. In the context of the highly generous French national health and social care system, which is easily accessible and does not require out-of-pocket payment, the French Health Ministry insists on rigorous techniques, including randomized protocols, to evaluate the impact of Housing First approaches in France.

**Method and design:**

A prospective randomized trial was designed to assess the impact of a Housing First intervention on health outcomes and costs over a period of 24 months on homeless people with severe mental illness, compared to Treatment-As-Usual. The study is being conducted in four cities in France: Lille, Marseille, Paris and Toulouse. The inclusion criteria are as follows: over 18 years of age, absolutely homeless or in precarious housing, and possessing a ‘high’ level of need: diagnosis of schizophrenia or bipolar disorder and moderate to severe disability according to the Multnomah Community Ability Scale (score ≤ 62) and at least one of the following three criteria: 1) having been hospitalized for mental illness two or more times in any one year during the preceding five years; 2) co-morbid alcohol or substance use; and 3) having been recently arrested or incarcerated. Participants will be randomized to receiving the Housing First intervention or Treatment-As-Usual. The Housing First intervention provides immediate access to independent housing and community care. The primary outcome criterion is the use of high-cost health services (that is,, number of hospital admissions and number of emergency department visits) during the 24-month follow-up period. Secondary outcome measures include health outcomes, social functioning, housing stability and contact with police services. An evaluation of the cost-effectiveness and cost-utility of Housing First will also be conducted. A total of 300 individuals per group will be included.

**Discussion:**

This is the first study to examine the impact of a Housing First intervention compared to Treatment-As-Usual in France. It should provide key information to policymakers concerning the cost-effectiveness and health outcomes of the Housing First model in the French context.

**Trial registration:**

The current clinical trial number is NCT01570712

## Background

Homelessness, defined as the absence of customary and regular access to a conventional dwelling or residence [1], has been recognized as a growing social and public health problem in developed countries since the end of the 1980s. An estimated 100,000 people live on the streets in France^a^. The number of homeless people is expected to rise in the current economic context of growing inequalities with potentially dramatic effects on health [2,3]. Indeed, homeless people in most Western countries have limited access to appropriate care for their complex health care needs [4]. Adherence to treatment and continuity of care are often poor [5]. They also have a greater risk of engaging in health-damaging behaviors, including tobacco, alcohol and drug dependence in particular [6,7]. In addition, it has been estimated that more than one in four homeless people suffer from serious mental illnesses, such as schizophrenia or bipolar disorder [8-11]. Providing care to those homeless people with severe mental illness is particularly challenging [12]: they are more likely to remain homeless for longer periods of time and to require more health support (that is, they tend to be in poorer physical health and to have more substance abuse co-morbidity) and more social support (that is, they have less social support, they encounter more barriers to employment and they have more contact with the legal system) than homeless people who do not suffer from mental illness [13,14].

Over the last several decades, two main approaches have been investigated regarding treatment for homeless people with mental illness in the United States and Canada [15,16]. The Treatment First model, also called Continuum of Care, requires that homeless people first address mental health issues and/or drug misuse, moving progressively up a ‘staircase of transition’ before finally accessing independent housing [17]. In contrast to this model, which predominates in many European countries, including France, the Housing First model reverses this sequence by offering immediate access to stable housing [18]. Individuals can exercise some degree of choice regarding the location and type of housing they receive. No pre-conditions, such as being stabilized on medications or bringing substance abuse under control, are necessary. In addition, homeless people in the Housing First model access support from a multidisciplinary team, following a well-defined intensive case management (ICM) program for people with moderate needs (that is, participant/staff ratio = 20:1; availability: five days/week, eight hours/day) or assertive community treatment (ACT) for those with greater needs (that is, participant/staff ratio = 10:1; availability: seven days/week, twenty-four hours/day).

Recent reviews of the literature suggest that in North American contexts, the Housing First model is particularly appropriate for homeless people with mental illness [15,16]. Compared to Treatment First or Treatment-As-Usual programs, Housing First programs report greater residential stability and fewer arrests, greater control over drug and alcohol use, better health outcomes and well-being, and lower residential and health costs [18-24]. However, is the Housing First model applicable in European contexts [25]? To our knowledge, no randomized studies have explored the impact of the Housing First model in Europe. Given the important differences in health and social care provision [26,27], such models need to be tested and evaluated in these highly generous care contexts, such as in France, before implementation on a larger scale. In line with international recommendations on poverty policies and programs [28,29] and a recent French national report on health and homelessness [30], the French Ministry of Health recommends applying rigorous techniques, such as randomized evaluations, to develop and test the Housing First model with real-world problems faced by the homeless in France. We thus designed a prospective randomized trial to assess the impact of a Housing First intervention in comparison with Treatment-As-Usual (that is,, control group receiving existing supports and services in each site) on health outcomes and costs for homeless people with severe mental illness. This project is supported both by private (less than 10%) funding sources and public funding sources, issued from different policymakers in charge of medical, social, housing and research services.

## Methods and design

### Study objectives

The primary objective is to assess the impact of a Housing First intervention in comparison with Treatment-As-Usual on the use of high-cost health services (that is, number of hospital admissions and number of emergency department visits) over a 24-month period by homeless people with severe mental illness in France.

The secondary objectives are: (1) to assess the impact of the Housing First intervention on the number of days in hospital and health outcomes (that is, mental health, physical health, alcohol and substance use, quality of life and recovery), adherence to medication, social functioning, housing stability and contact with legal services; (2) to measure the cost effectiveness and cost utility of the Housing First intervention in comparison with Treatment-As-Usual; and (3) to describe changes in professional culture and practices of the different stakeholders involved in the Housing First program, including policymakers, professionals and users, using qualitative research methods.

### Study site and population

The study is being conducted in four major cities in France: Lille, Marseille, Toulouse and the capital city, Paris. The inclusion criteria are as follows: over 18 years of age; absolutely homeless^b^ or precariously housed^c^; living in the city in question for more than six months and intending to stay in that same city for the coming two years; possessing a ‘higher’ level of need, defined as having a diagnosis of schizophrenia or bipolar disorder according to Diagnostic and Statistical Manual of Mental Disorders, fourth edition (DSM-IV-TR) criteria [31] and moderate to severe disability according the Multnomah Community Ability Scale (score ≤ 62) [32,33] and meet at least one of the following three criteria: 1) having been hospitalized for mental illness two or more times in any one year over the last five years, or 2) having co-morbid alcohol or substance use, or 3) having been arrested or incarcerated over the last two years; being covered by French government health insurance including free state aid; and speaking French. Exclusion criteria included the following: being considered unable to provide informed consent [34]; having dependent children; pregnancy; or a DSM-IV Axis I diagnosis other than schizophrenia or bipolar disorder.

### Study design and procedure

**Figure 1:**
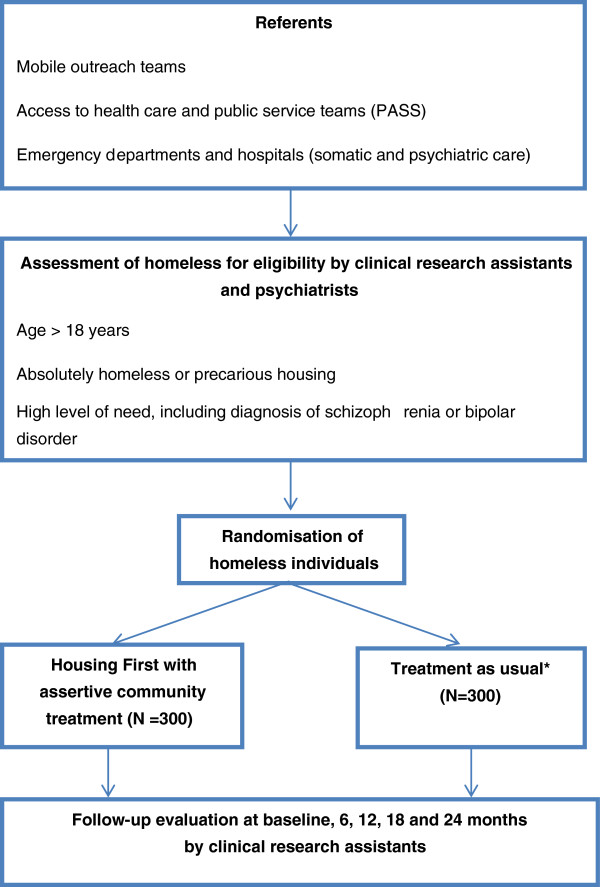
Flowchart of the study protocol.

The present study is a 24-month, prospective, randomized, controlled, open-label, and multi-site study, based on a mixed approach combining quantitative and qualitative methods. Subjects are referred from a wide variety of services including mobile outreach teams [35], community mental health services, general hospitals and access to health care and public services teams (Figure 1). Trained research assistants check eligibility criteria within 24 hours of referral. The results from the Mini International Neuropsychiatric Interview are given by a psychiatrist [36]. The assistants describe the study, respond to any questions the candidates may have and obtain written informed consent. Participants are then randomly assigned to either the Housing First intervention (Group 1) or Treatment-As-Usual care (Group 2). Randomization is stratified by site and a computer-generated, randomized list is created using a permuted block design. Quantitative data are collected during face-to-face interviews by the trained research assistants at five different points in time: at randomization (baseline; T0) and then at 6 (T1), 12 (T2), 18 (T3) and 24 months (T4) after randomization. All data are collected using netbook (small inexpensive laptop computers) computer-assisted interviewing and transferred to a highly secure central database without using internet (EpiConcept®) http://www.epiconcept.fr/.

The qualitative approach is based on a generalizable study design (that is, to determine whether the Housing First model evaluated in this study may be transposed to other real-life settings in the French context) [37] using validated methods for assessing health care including observational data from participants, interviews and focus groups [38,39]. The persons who carry out the research will enter the culture of those they study and experience the events in the way in which the participants experience them. In addition to direct observation, in-depth interviews and focus groups with policymakers, professionals and users will be performed. Interviews and focus groups will be recorded and transcribed verbatim. These methods will be administered at (baseline; T0) and then at 6 (T1), 12 (T2), 18 (T3) and 24 months (T4).

The assessment schedule is presented in Table 1.

**Table 1:** Assessment schedule

	**Screening**	**Evaluation**				
		**Baseline**	**6 months**	**12 months**	**18 months**	**24 months**
Eligibility criteria	x					
Informed consent	x					
Health care use			x	X	x	x
MCSI		x	x	x	x	x
CGI		x	x	x	x	x
MINI		x	x	x	x	x
MARS		x	x	x	x	x
SF-36		x	x	x	x	x
S-QoL 18		x	x	x	x	x
EuroQoL 5D		x	x	x	x	x
RAS		x	x	x	x	x
MCAS	x	x	x	x	x	x
*Ad hoc* questionnaires		x	x	x	x	x
Cost analysis			x	x	x	x

*CGI*, clinical global impression; *Health care use*, number of admissions to hospital, total days in hospital and number of emergency department visits; *MARS*, medication adherence rating scale (MARS); *MCAS*, Multnomah community integration scale *MINI*, mini international neuropsychiatric interview, sections K and L; *MCSI*, modified Colorado symptom index; *RAS*, recovery assessment scale; *SF-36*, medical outcomes study 36-item short form health survey; *S-QoL 18*, schizophrenia quality of life questionnaire.

### Groups

• Group 1 (‘Housing First’): participants are provided with immediate access to independent housing. However, they must agree to accept a maximum of 30% of their income paid directly as rent and receive home visits at least once a week by program staff. In addition, they have access to a recovery-oriented, assertive community treatment (ACT) team [40] which includes a doctor, a nurse, a social worker and a peer worker. In particular, this team provides case management, mental health services, employment and housing assistance, substance abuse services and other services to allow the individual to live successfully in the community.• Group 2 (‘Treatment-As-Usual’): homeless individuals receive usual care, that is, pre-existing programs and services in the cities of Lille, Marseille, Paris and Toulouse.

### Evaluation criteria

#### Primary objective

The primary evaluation criterion is the use, during the 24-month follow-up period, of more costly health services, as measured by the number of hospital admissions and number of emergency department visits.

#### Secondary objectives

Mental health is specifically assessed using the Modified Colorado Symptom Index (MCSI) [41] and the Clinical Global Impression (CGI) [42]. The MCSI contains 14 items which evaluate how often in the past month an individual has experienced a variety of mental health symptoms, including loneliness, depression, anxiety, and paranoia. Higher scores indicate a greater likelihood of mental health problems. The CGI rates the overall severity of any mental disorder, on a scale of the overall current severity of symptoms from 1 (healthy, not ill) to 7 (severely ill). The CGI is sensitive to change and correlates well with changes assessed with more complex scales [43,44].

Alcohol and substance use is assessed using sections K and L of the Mini International Neuropsychiatric Interview (MINI) [36]. The MINI is an abbreviated, structured diagnostic interview that determines the presence or absence of diagnoses of dependence on and/or abuse of alcohol and/or the more frequently used or more problematic drugs, and whether the diagnosis is current (preceding 12 months) and/or a lifetime diagnosis (anytime in life - may or may not be current).

Adherence is assessed with the Medication Adherence Rating Scale (MARS) [45], a 10-item, multidimensional, self-reporting instrument describing three dimensions: ‘medication adherence behavior’, ‘attitude toward taking medication’ and ‘negative side-effects and attitudes to psychotropic medication’. A high score correlates with a higher likelihood of medication adherence.

Global physical and mental health status is assessed using the 36-item Short Form Health Survey (SF-36) of the Medical Outcomes Study [46]. The SF-36 is a self-administered questionnaire consisting of 36 items describing eight dimensions: physical functioning, social functioning, role-physical problems, role-emotional problems, mental health, vitality, bodily pain, and general health. Two composite scores can be calculated: the physical composite score and the mental composite score. Each dimension is scored within a range from 0 (poor health status) to 100 (good health status).

Quality of life is assessed using the S-QoL 18 [47], which is a self-administered, multidimensional questionnaire developed and validated for the specific assessment of quality of life in patients with mental disorders [47,48]. The S-QoL 18 comprises 18 items describing eight dimensions: psychological well-being, self-esteem, family relationships, relationships with friends, resilience, physical well-being, autonomy, and sentimental life. It also generates a global score. Dimension and index scores range from 0, indicating the lowest quality of life, to 100, the highest quality of life.

Health status is assessed using the EuroQoL 5D [49], a standardized instrument for use as a measure of health outcomes, providing a simple descriptive profile and a single index value. This self-administered questionnaire measures five dimensions: mobility, personal care, routine occupations, pain and discomfort, and anxiety and depression. Each dimension has three levels: no problems, some problems, and severe problems.

Recovery is assessed using the Recovery Assessment Scale (RAS) [50], which measures various aspects of recovery from the perspective of the consumer, with a particular emphasis on hope and self-determination. This self-administered instrument comprises 24 items, exploring five domains: personal confidence and hope, willingness to ask for help, goal and success orientation, reliance on others, and no domination by symptoms. A higher score indicates better recovery.

Social functioning is assessed using the Multnomah Community Integration Scale (MCAS) [32,33]. The MCAS is a 17-item instrument that measures the degree of functional ability of adult clients who have severe and persistent mental disorders and who live in the community. Items are grouped into four categories: 1) interference with functioning; 2) adjustment to living; 3) social skills; and 4) behavioral problems. Higher scores indicate more severe disability.

The following parameters are also recorded by research assistants using *ad hoc* questionnaires elaborated by the steering committee composed of economists, psychiatrists, psychologists, social workers and sociologists: gender, age, education level, social minimums and administrative situation, employment status, social network, health events, housing stability, contact with legal services, use of social services (for example., emergency shelters, transition shelters, stabilization shelters, supportive housing, and hostels) and experience of violence.

In the cost analysis, direct and indirect costs are measured during the 24-month follow-up period. Direct costs comprise the costs related to medical/health, legal, housing, and social services (that is, hospital days; emergency department visits; outpatient visits; use of substance abuse treatment centers; legal services, including days detained in jails and prisons; days in respite, shelter, and other housing; and case management) and indirect costs mainly related to loss of productivity [51,52]. Data are collected using national administrative, social and medical databases; medical records; structured interviews with research participants and the standardized Short Form-Health and Labor questionnaire (SF-H&L) [53]. The SF-H&L inquires about productivity losses that are caused by health problems in general: absenteeism from paid work, production losses without absenteeism from paid work and hindrance in the performance of paid and unpaid work.

### Sample size

Sample size was calculated to obtain 90% power, to detect a 20% difference in the use of costly health care services (reference points = 3.6 for the number of hospitalizations and 5.7 for the number of emergency department visits [54]) at 24 months between the two groups. With a significant *P*-value of 0.05, these calculations showed that a total of 250 individuals per group was required; allowing for a potential 20% of patients being lost to follow-up, a total of 600 will need to be included, that is, 150 at each site.

In the qualitative study, an extensive review of relevant literature and theory will guide sample selection from the four sites to capture diversity of experiences in the Housing First program. Adjustment will be performed in the course of the study to respond to unanticipated patterns of subgroups of experiences, which may need increased representation in the sample.

### Strategies for reducing attrition

Several strategies are used to minimize loss to follow-up. First, the study has been designed so that research assistants can spend a large amount of time tracking participants. Research assistants will receive specific training by existing mental health outreach teams to establish quality working alliances with homeless individuals. At baseline, research assistants underline the importance of participating in the follow-up interviews. Participants are asked to provide information about their families, friends, habits, service providers, and so forth that could help locate them. In addition, participants of the control group will receive €21 worth of food coupons for each interview.

### Statistical analysis

The data will be summarized using the mean, median, standard deviation and range for quantitative data and counts and frequencies for categorical data. The analyses on the primary and secondary criteria will be performed on the intent-to-treat population (that is, comparison of patients in the group to which they were originally randomly assigned). We will conduct analyses for each of the outcomes separately (that is, number of hospital admissions and number of emergency department visits). In addition, complementary per protocol analyses will be performed (that is, comparison of patients who completed the treatment originally allocated). Finally, missing data will be handled where possible (for example, quality of life and recovery) using multiple imputations; sensitivity analysis will be conducted.

Comparisons between the two groups for each outcome will be performed using Student’s *t*-tests or Mann–Whitney-Wilcoxon tests for quantitative or ordinal variables, and chi-square or Fisher’s exact tests for frequencies. Non-parametric tests will be used for data that is not normally distributed. Multivariate analyses will be performed primarily using negative binomial regression models for the number of admissions to hospital and a gamma distribution for the number of days in hospital, adjusting for the length of follow-up. Multiple imputation techniques for missing data will be discussed. Moreover, linear regression models will be used. The dependent variable will be the primary evaluation criterion (that is, the use of the health care system). Explanatory variables will be selected among those for which the *P*-value is less than or equal to 0.20 in univariate analysis, and described in the literature as being associated with use of the health care system. The parameter ‘site’ will be considered as a confounding factor. The results will be presented in the form of standardized beta coefficients.

Statistical significance is defined as *P* > 0.05. Statistical analyses will be performed using SPSS Statistics for Windows, Version 17.0 ( SPSS Inc., Chicago, IL, USA).

### Cost analysis

An economic evaluation will be carried out from a societal perspective and according to the French guidelines for costing in economic evaluations [55,56].

Direct costs will be calculated by weighing the use of health, legal, housing, and social services by unit cost estimates obtained from French national data sets and reports, for example, from the national database for outpatient visits and Health Ministry hospital reimbursement reports for inpatient care and day-clinic treatment. Two methods of calculating indirect costs will be examined: the traditional human capital approach and the developed friction cost method [57,58]. Benefits will be assessed in terms of recovery (RAS) [59] and health status (EuroQoL 5D). Efficiency will be evaluated using the Incremental Cost-Effectiveness Ratio (ICER), defined as cost per recovery point or QALY gained with ‘Housing First’ versus ‘Treatment-As-Usual.’

### Qualitative analysis

Analysis will be essentially based on a taxonomic process. Content analysis of interviews and focus groups will be performed by sociologists who are skilled in textual analysis, complemented by a computerized textual analysis. Data will be sorted to give coherent categories of experience, drawing not only on the initial theoretical framework but also on theoretical concepts that emerge from the data. Special attention will be given to the evaluation of the institutional and political dynamics involved in traditional rehabilitation approaches, the trajectories and energies in interplay in the recovery-based Housing First program, the relationship between citizenship and processes of rehabilitation and recovery and, finally, the conditions for implementing the program on a larger scale should the results prove positive.

### Ethical principles and safety

The study is designed and carried out in accordance with the principles of the Declaration of Helsinki, sixth revision [60]. The patients are provided with both oral and written information regarding the study prior to obtaining their informed consent. The local ethics committee (Comité de Protection des Personnes Sud-Méditerranée, France) approved this study, which is registered with the international standard randomized control trial number (NCT01570712).

## Discussion

This study is the first large-scale attempt to assess the applicability of the Housing First model on homeless people with serious mental illness in France. Several studies have provided evidence regarding the effectiveness of the Housing First model in North American contexts [15,16,21,23,54,61]. However, the extent to which the Housing First model could be replicated in France needs to be rigorously investigated [30]. The management of health care and housing for homeless people with mental disorders in France is significantly different from health and social care services in the North American contexts where the aforementioned studies were carried out. Generalization from the findings and expected benefits of these studies of North American contexts to the French context would be hazardous. Indeed, the health care policy of universal coverage in France, as opposed to nonuniversal coverage, may have a significant impact on the management of care for homeless people with mental disorders [26]. A recent study has suggested that access to care for homeless people may be higher in France, with its near-universal coverage and free mental health system [5]. In addition, mobile mental health outreach teams including doctors, nurses and social workers have been created since 2005 to ensure the provision of health, mental health and social care for ‘hard to reach’ homeless individuals with and without severe mental disorders [35]. Finally, a range of social aid and housing services can be easily accessed by all citizens in France who meet appropriate criteria, including direct financial support, disability allowances and housing assistance. Of particular note is the 2007 legislation introducing a theoretically enforceable right to housing for all citizens. Thus, services provided in the current study for the Treatment-As-Usual group are far more extensive than in previous studies in other national contexts. The present study is therefore of great importance to confirm whether these previous findings can be generalized to the French-specific context.

A major strength of the current project is the proposition of a rigorous evaluation of two policy options (‘Housing First’ versus ‘Treatment-As-Usual’) with regard to answering the specific needs of the homeless in France. Although randomized experiments have become increasingly popular in recent years, medico-social experiments in real-life contexts using a randomized project design as in the current study are still relatively rare [29]. The advantage of this approach is that the results are more representative of everyday practices and provide significant evidence regarding the feasibility of using the Housing First intervention as part of ongoing routine care management in the French context. This thus increases the generalizability of the results and supports implementation [62]. Another advantage of randomized experiments is the interplay between theory and experimental research resulting from the close collaboration between researchers and implementers/policymakers [63]. In the present study, special attention has been paid to this sort of collaboration, involving a large number of professionals working closely together, including policymakers at national and local levels; researchers from multiple domains, including psychiatrists, public health professionals, sociologists and economists working within an independent national scientific committee (Institut de Recherche en Santé Publique); and health and social care professionals (doctors, nurses, social workers and peer workers). Finally, this study has been granted a significant budget, drawn from public funds with large teams of professionals, enabling the implementation of this complex intervention. Ambitious experiments as in the present study are all the more likely to be useful for influencing national policy in these areas [64].

Another strength and originality of the present study is the proposition of a qualitative approach in addition to quantitative methods to investigate the complex health and social issues surrounding the current Housing First program. Qualitative research is particularly well suited to understanding complex phenomena, uncovering links among concept and behaviors, and generating and refining theory applicable to specific contexts [65]. In addition, mixed methods approaches, including qualitative and quantitative approaches, provide unique contributions to outcomes research [65,66].

This study has several limitations. Even with the large overall sample size in this multicenter study, the sample is not representative of the homeless population as a whole. The inclusion criteria used in the present study only concern a particular section of the homeless population: those with high needs and severe mental illness. Generalization from the findings of this study to other homeless people would be hazardous. However, should Housing First prove to produce positive results in the French context, it is arguable that this may well remain true for less vulnerable individuals. Further work is needed to confirm this hypothesis, especially in populations with moderate needs and without mental disorders.

A further challenge for the present study will be to retain participants at the time of follow-up. Previous longitudinal studies on homeless individuals have managed to retain only 60 to 85% of participants over follow-up periods of 18 to 36 months [14]. As previously explained, attempts to improve follow-up rates in the current study will include using food coupons, obtaining contact details of significant others to help with locating participants and explaining to participants the importance of conducting follow-up.

Finally, we cannot exclude a series of limitations, such as potential Hawthorne effects (that is, individuals from the Housing First group may be conscious of being observed or indeed benefit from being observed, which may induce them to change their behavior for the duration of the experiment, or indeed for longer) or John Henry effects (individuals from the Treatment-As-Usual group may feel offended to be a comparison group and react by altering their behavior). Contamination bias between individuals in the two groups is also a possibility, although less likely because the number of patients included in the present study is relatively small compared to the total number of individuals seeking housing at each site. The qualitative analyses used in the present study should help us to analyze these different phenomena and take them into account when interpreting results.

## Trial status

The study started recruiting participants in August 2011, and the recruitment is ongoing.

## Endnotes

^a^Source: http://www.emmaus-france.org.uk, 2007.

^b^Absolutely homeless is defined as having had no fixed place to stay for at least the past seven nights with little likelihood of finding a place in the coming month.

^c^Precariously housed is defined as being housed in single room occupancy, rooming house, or hotel/motel as a primary residence AND in the past year having experienced two or more episodes of being absolutely homeless OR one episode of being absolutely homeless that lasted for more than four weeks in the preceding 12 months.

## Competing interests

The authors declare that they have no competing interests.

## Authors’ contributions

AT, CF, VG and PA conceived and designed the study, including the statistical elements and power calculation. The study protocol and manuscript have been drafted by AT, CF, LB and PA. The study protocol and manuscript were critically reviewed by VG, CL, BV, PR, TG, BF, TA and CL. All authors read and approved the final manuscript.
